# Can verbal autopsies be used on a national scale? Key findings and lessons from South Africa's national cause-of-death validation study

**DOI:** 10.1080/16549716.2024.2399413

**Published:** 2024-09-13

**Authors:** Monique Maqungo, Nadine Nannan, Beatrice Nojilana, Erin Nichols, Diane Morof, Mireille Cheyip, Chalapati Rao, Carl Lombard, Jessica Price, Kathleen Kahn, Lorna J. Martin, Francois Bezuidenhout, Ria Laubscher, Chodziwadziwa Kabudula, Tracy Glass, Oluwatoyin Awotiwon, Nesbert Zinyakatira, Noluntu Funani, Jané Joubert, Debbie Bradshaw, Pamela Groenewald

**Affiliations:** a Burden of Disease Research Unit, South African Medical Research Council, Parow Valley, Western Cape, South Africa; b National Center for Health Statistics, U.S. Centers for Disease Control and Prevention, Hyattsville, MD, USA; c U.S. Public Health Service Commissioned Corps, Rockville, MD, USA; d Division of Global HIV & TB, Centers for Disease Control and Prevention, Durban, South Africa; e Division of Global HIV/AIDS and TB, Centers for Disease Control and Prevention, Pretoria, South Africa; f Department of Global Health, Research School of Population Health, Australian National University, Acton, Australian Capital Territory, Australia; g Biostatistics Research Unit, South African Medical Research Council, Parow Valley, Western Cape, South Africa; h SAMRC/Wits Rural Public Health and Health Transitions Research Unit (Agincourt), School of Public Health, University of the Witwatersrand, Johannesburg, South Africa; i Division of Forensic Medicine and Toxicology, Faculty of Health Sciences, University of Cape Town, Cape Town, South Africa; j GeoSpace International, Pretoria, South Africa; k Health Impact Assessment Unit, Western Cape Department of Health, Cape Town, South Africa; l School of Public Health and Family Medicine, Faculty of Health Sciences, University of Cape Town, Cape Town, South Africa

**Keywords:** Cause of death, cause-specific mortality fractions, Civil Registration and Vital Statistics, verbal autopsy, South Africa

## Abstract

**Background:**

Verbal autopsy (VA), though imperfect, serves as a vital tool to determine cause-of-death, particularly for out-of-facility deaths, but challenges persist in integrating VA into Civil Registration and Vital Statistics systems.

**Objective:**

To describe the challenges and successes of collecting a national sample of verbal autopsy interviews in South Africa to obtain the cause of death profile in 2017/18.

**Methods:**

We recruited next of kin from 27 randomly selected sub-districts (10.5%) across South Africa between September 2017 and April 2018. Trained fieldworkers conducted face-to-face interviews using the WHO2016 VA instrument, with physicians certifying underlying causes of death. Feasibility was evaluated based on response rates, participation, and data quality.

**Results:**

Of the total 36,976 deaths registered, only 26% were identified during recruitment, with a 55% overall response rate for VA interviews. Physician-reviewed VA data were deemed of good quality for assigning underlying causes of death in 83% of cases. By comparing cause-specific mortality fractions, physician-reviewed VA identified 22.3% HIV/AIDS and InterVA-5 identified 18.5%, aligning with burden of disease estimates, while Statistics South Africa reported 4.9% HIV/AIDS.

**Conclusions:**

The study demonstrated the feasibility of using VA on a national scale, but immense challenges in identifying and recruiting next of kin highlight the importance of formalising VAs within the country’s death notification system.

## Paper context

**• Main findings**: Next of kin of 9 730 decedents were approached at the time of registration of death and 55% consented to be approached later and agreed to do a VA interview by a trained field-worker; 83% of physician-reviewed VA data were considered high-quality for determining underlying causes and 22.3% of all the deaths were due to HIV/AIDS, much higher than the proportion reported in the national statistical office.

**• Added knowledge**: Implementing the VA on a national scale was achievable but significant challenges in recruiting next of kin, emphasising a need to formalise VAs within the country’s death notification system.

**• Global health impact for policy and action**: Accurate cause-of-death data are crucial for policymakers to make informed decisions about the country’s health system and could be supported by using VAs, particularly for the deaths that occur outside health facilities.

## Background

Reliable, continuous, and timely mortality and cause-of-death (COD) data are essential for improving health and population policies and supporting countries to respond to emerging health threats and epidemics [1,2]. The importance of recording vital events is well recognised, and Civil Registration and Vital Statistics systems (CRVS) that provide continuous data on births, deaths and COD are now seen as a fundamental data source to monitor the Sustainable Development Goals [3]. Fifteen of the goals require CRVS data, and 14 of the indicators require cause-specific mortality [3]. However, in many low- and middle-income countries, low infrastructure and fiscal investments in civil registration systems have resulted in countries having low levels of death registration and limited or no medical certification of COD.

Verbal autopsy (VA) is a method used to collect and analyse COD information based on an interview conducted with the next of kin or close caregiver about the illness and circumstances leading up to death. Although VA is an imperfect tool to determine the COD, it is often the only population-health option in identifying the COD for out-of-facility deaths [2]. It has been suggested that in countries with limited access to health services or medical officers, more extensive use of VA could help fill the information gap [4]. In 2017, de Savigny and colleagues [5] presented a detailed systematic view of how VA can be integrated into CRVS. The Bloomberg Philanthropies Data for Health Initiative conducted a structured mapping exercise to identify and map current system responsibilities and data flow for CRVS systems in 16 countries [6]. Both studies revealed the challenges involved with integrating VA into CRVS systems. To date, we are not aware of any country that has fully integrated VA into CRVS.

In 2005, the first international technical standards and guidelines for VA were introduced [7]. The 2007 VA standard instrument includes separate questionnaires for three age groups with a defined VA list (Annexure 1, Table 2) of causes and corresponding codes from the 10th revision of the International Statistical Classification of Diseases and Related Health Problems (ICD-10) [8]. In 2016, questions were added or edited to reach full compatibility with the available automated analysis methods to reduce clinician burden in reviewing questionnaires [9].

South Africa has made great strides in increasing geographic coverage of death registration [10] following integration of the ‘homeland’ areas and the enactment of the Births and Deaths Registration Act of 1992 [11]. Deaths must be registered within 72 h. Recent estimates indicate that completeness for persons above the age of 2 years is over 90% [12]. Despite these improvements in death registration, there are still concerns about the quality of data relating to COD. These include a high proportion of deaths with ill-defined causes (13%), with an additional 13% having a COD that is not valid as an underlying cause in 2016, under-reporting and misclassification of HIV deaths, and an inaccurate profile of injury deaths [13]. The extent of these problems differ at district and national levels [14], and arise from a combination of certifying medical doctors not having access to a full medical history at the time of certification, poor certification practices, and some of the deaths being certified through an affidavit completed by a headman (traditional area administrator) rather than a medical certificate. Although South Africa has three health and demographic surveillance sites [15–17] as well as a Child Health and Mortality Prevention Surveillance (CHAMPS) site [18] that routinely use VA to track COD, there has never been national implementation of VA.

The South African National Cause-of-Death Validation Project aims to validate the CRVS COD information by collecting VA, medical, and forensic pathology record data on the COD for a national sample of deaths and then compare this with information recorded in the CRVS system. The initial data analysis of the VAs demonstrated the feasibility of setting up a national collection of VA data [19]. This paper presents the methodology and findings related to VAs and highlights important issues for countries considering using VAs within a national initiative.

## Methods

We conducted a cross-sectional study that collected data for a fixed-period census of deaths that occurred in a nationally representative sample of health sub-districts in South Africa during parts of 2017 and 2018, using a probability proportional to population size sampling strategy. A detailed methodology is reported elsewhere [19].

In the first phase of data collection, funeral practitioners who serviced the sampled area and were designated by the Department of Home Affairs as official registration agents were recruited to the study. Since the Protection of Personal Information Act (POPIA) [20] of 2013 precludes Department of Home Affairs or the Department of Health from sharing personal information of the next of kin without their consent, it was necessary to work through funeral practitioners and Department of Home Affairs officials as intermediaries to obtain consent to participate in the study. They were asked to share information about the study with the family or informant and requested permission to be contacted by researchers.

Trained fieldworkers contacted consenting informants and arranged VA interviews at least 3 months after the date of death. In addition, the medical records and forensic pathology records were collected from facilities serving the selected areas. The VAs and records were reviewed by medical doctors trained in medical certification of COD. In the final phase of the study, the underlying COD reported in the CRVS system will be validated against the underlying cause identified through the highest level of evidence collected in the study for each decedent.

### Sampling and sample size determination

**Figure 1.**
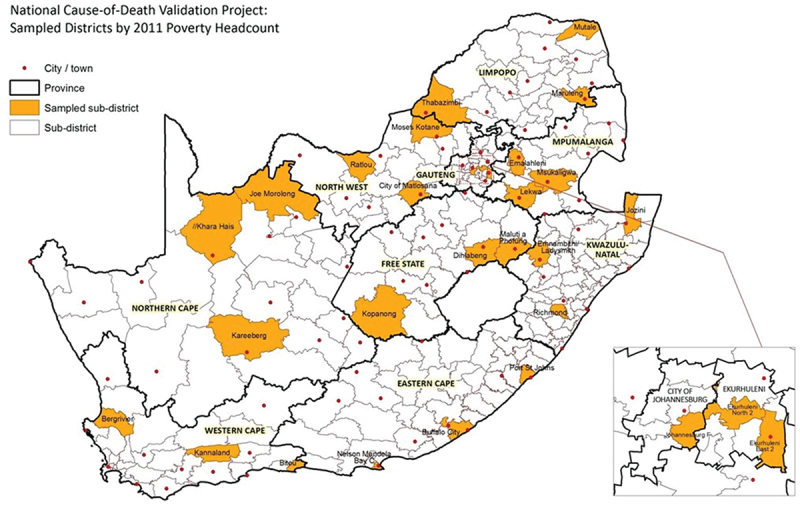
Map of selected health sub-districts and provincial boundaries, South African national cause of death validation project 2017/18.

The study population comprised registered deaths from 1 September 2017 until 13 April 2018. We randomly selected 27 sub-districts (Figure 1) for inclusion of all the registered deaths that occurred over a 3-month period in these areas. This accounted for 10.5% of the total population with a median size of 116 000 population per selected sub-district. Using pseudo stratification, three sub-districts were selected from each province according to socio-economic status based on the population size. Due to the true frequency of specific causes of death, the error rate and the extent of clustering that would arise through geographic correlations, sample size determination was not feasible. Instead, scenarios were considered based on the estimated COD profiles, considering the correction factor to be 50%, allowing for a design effect of 2 and a response rate of 85%. It was determined that a sample size of 13 000 deaths would produce a 2–3% precision on the correction factor for HIV/AIDS; 4–5% for cerebrovascular disease; and 7% for diabetes mellitus and interpersonal violence.

During the first 6 weeks, we realised that recruitment of next of kin was extremely challenging. The study period was therefore extended to 8.5 months after which 9 730 informants were approached, with 65% consented. The study protocol was amended to increase the sample size by including deaths of decedents who died in a health facility or who were referred to forensic pathology services.

### Data collection

We modified the WHO 2016 questionnaire to start with the narrative which outlines the events leading up to the death in order to establish rapport during the interview and prevent the respondent repeating the story of events after having provided these details through the questions. Minor changes were made to clarify questions related to maternal deaths and to injuries. We used the Open Data Kit (ODK) dictionary to set up the questionnaire in KoBotoolBox, an online data collection tool [21] with translations into eight local languages. A hard copy of the information sheet was given to the respondents to sign and keep. The KoBotoolBox data collection form made provision for digital signatures.

A team of 84 fieldworkers, selected on the basis of having a university degree or a school-leaving certificate with adequate fieldwork experience, were trained in conducting VAs. Each fieldworker received a tablet containing the data collection tools and fieldworker manual. The fieldworkers were also trained to capture photographic images of de-identified medical and forensic records [22]. During the training, continuous assessments identified issues that required further input for the trainees.

### Identification of cause of death

A total of 51 physicians with clinical experience in the South African Public Health Service reviewed the collected information and identified the underlying COD. The physicians were orientated to the VA instrument, how to review completed VA questionnaires and use of the customised data collection tools. In order to standardise COD information, physicians were trained in the principles of ICD-10 medical certification of COD [8] and were given standard operating procedures for record reviews.

Anonymised responses to the VA interviews were batched into 40 and independently reviewed by two physician reviewers. Once the batch was completed, quality assurance reviewers evaluated the submissions and identified cases where reviewers assigned different underlying COD. In these cases, re-examination and discussion were necessary to reach consensus.

Physician reviewers also assessed the quality of the clinical information available to identify the COD. They used a subjective score on a scale of 1 (very poor) to 5 (excellent) based on the consistency and coherence of clinical information provided in the narratives and the questionnaire responses. In addition, they scored the sufficiency of the information, on a scale of 1 (very poor) to 5 (excellent), to make a decision about the underlying COD.

### Data processing and analysis

We used IRIS V5.8.1 automated software [23] to code the text from the medical certificates and select the underlying COD in ICD-10. We extended the dictionary of medical terms that was developed for IRIS for the Western Cape local mortality surveillance system [24] by adding terms that were commonly used by physicians. Two researchers trained in ICD-10 coding and a co-principal investigator manually coded records that were rejected by IRIS. In addition to the physician reviewers’ assessment of the information, the quality of the physician reviewed COD data was assessed using ANACONDA software [25]. It checks for biological implausible causes of death based on age and sex. Furthermore, it identifies unusable codes (impossible as underlying causes of death, intermediate causes of death, immediate causes of death, insufficiently specified causes, ill-defined symptoms) for the underlying cause of death.

The cause-specific mortality fractions (CSMF) based on the VA data reviewed by physicians was compared with that from the COD data reported by Statistics South Africa Stats (Stats SA) [26] based on the registered deaths (death notification forms including the medical certificate of cause of death). Stats SA code the information written on the medical certificate of cause of death to ICD-10 and uses the Automated Classification of Medical Entities (ACME 2011) in addition to IRIS to identify the underlying cause of death [26]. The COD data from both sources were aggregated to the South African National Burden of Disease (SA NBD) list [27] (shown in Annexure 1) which has been developed to suit the characteristics of the country’s disease profile and the quality of routine data.

The CSMF derived from the physician reviewed VAs were compared with those derived from the InterVA-5 algorithm [28] according to the VA list of conditions [9]. In this article, the VA results differ slightly from the preliminary findings, because some changes were made to the VA data through additional data cleaning. In addition, we have calculated the InterVA-5 CSMF by aggregating the likelihoods of causes (for up to three conditions for each case) according to the InterVA-5 guidelines compared with the profile provided in the previous report [19] which was based on the frequency of the most likely cause for each case. Approximate 95% confidence intervals (CI) have been calculated assuming the proportions follow a binomial distribution.

### Ethical consideration and permissions

Ethical clearance for research involving human participants was obtained from the SAMRC Ethics Committee, and the Health Research Ethics Committees at provincial health facilities. This project was reviewed in accordance with Centers of Disease Control and Prevention (CDC) human research protection procedures and was determined to be research, but CDC investigators did not interact with human subjects or have access to identifiable data or specimens for research purposes.

Funeral practitioners and Home Affairs officials identified all the deaths that occurred in the sample area within the study period. They recruited the next of kin, documented the contact details of consenting informants, and explained that researchers would contact them to arrange the VA interview.

During the fieldworker training the importance of confidentiality was explained. Project staff signed a confidentiality agreement from the undertaking to work ethically and ensure confidentiality. Personal information of the decedent was de-identified and a unique study ID allocated [19]. The study ID was applied to the individual patient records in multiple formats and anonymised datasets were created.

## Results

A total of 353 funeral practitioners and 95 Home Affairs offices were engaged to recruit next of kin (Table 1). Neither the next of kin nor their dwelling could be located for 560 decedents (8.9%). Out of the 5 756 located dwellings, there was a high response rate and 85.2% completed an interview resulting in an overall response rate of 55%. The main reasons for refusing an interview are shown in Table 2.

**Table 1.** Total number of funeral parlours, department of home affairs offices and next of kin recruited for the South African national cause of death validation project 2017/18 across the three subdistricts in each province.

Province	Offices		Informant			Next of kin	
	Funeral Parlour	Department of Home Affairs	Approached	Consented	Response rate	Interviewed	Overall response rate
Eastern Cape	70	11	1, 108	695	63%	575	52%
Free State	51	11	968	733	76%	656	68%
Gauteng	45	13	1, 552	626	40%	463	30%
KwaZulu-Natal	30	11	1, 578	1, 026	65%	884	56%
Limpopo	20	10	611	483	79%	377	62%
Mpumalanga	53	12	896	737	82%	610	68%
Northern Cape	28	6	907	545	60%	506	56%
North West	44	11	1, 846	1, 293	70%	1, 102	60%
Western Cape	12	10	264	220	83%	202	77%
South Africa	353	95	9, 730	6, 358	65%	5, 375	55%

**Table 2.** Response category and reason for refusal to participate in verbal autopsy interview (*N* = 5 756), South African national cause of death validation project 2017/18.

Response category and reason for refusal		Number	%
Dwelling located		5, 756	100.0%
Verbal autopsy conducted		5, 375	93.4%
Refused verbal autopsy Reason for refusal		381	6.6%
1	Too emotional about the death of their loved one to take part	182	3.2%
2	No interest in taking part in such a study	101	1.8%
3	Refused, either telephonically or face-to-face	47	0.8%
4	Respondent suspicious of all surveys	25	0.4%
5	Respondent cited a lack of time	15	0.3%
6	Information regarding the next of kin/informant incorrect	6	0.1%
7	Respondent indicated concern about legality of study	5	0.1%

**Figure 2.**
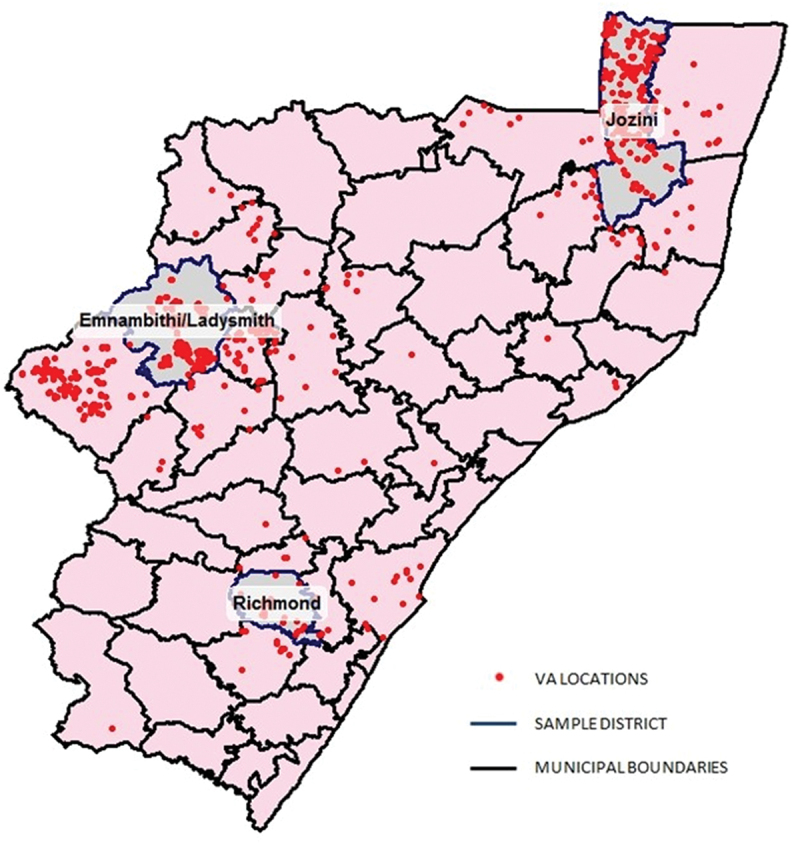
Map of KwaZulu natal showing selected health sub-districts and the address of verbal autopsy interviews conducted for South African national cause of death validation project 2017/18.

After completion of data collection, it was established that 36 976 deaths were registered during the study period and the VAs accounted for 15% of the target sample sub-districts. The geographic location where the interviews were conducted was often outside the sampled sub-districts, as the example of KwaZulu-Natal province in Figure 2 shows. The proportion of VAs falling within the boundaries of the designated sampled sub-district varied by sub-district. For instance, Jozini sub-district had a very good response rate with the majority of addresses falling within the target area. In contrast, the southern sub-district of Richmond had as many VAs falling outside of the designated area as compared to those which fell within the designated area.

The majority of VAs were conducted within the recommended time interval since the death occurred (83.0%), with a median time between death and the VA interview of 9.2 months. However, a small proportion (0.2%) were conducted less than 3 months since the death occurred, and 16.7% were conducted more than 12 months since the death occurred (median interval of 13.6 months).

Table 3 shows an assessment of the quality of VA information. The information was subjectively assessed on a scale of 1 = (poor) to 5 = (excellent) based on the clinical consistency of information in the narrative and the questionnaire responses, as well as the sufficiency of the VA information for purposes of certification of COD. The physicians scored the majority (81.6%) of the VAs as good quality (score 3–5), while less than 13.2% of the records were assessed as poor-quality information (Table 3). About 66% of VAs had sufficient information to assign the underlying COD (score 3–5). Out of the 5 086 cases with complete information on both criteria, 61.3% had exactly the same score for quality and sufficiency. The kappa statistic was 0.45 (95% confidence interval (CI): 0.44–0.46), indicating a moderate level of agreement between the two dimensions.

**Table 3.** Physicians’ assessment of quality and sufficiency of information from verbal autopsy (*N* = 5,375) for the South African national cause of death validation project 2017/18.

Score	What was the quality of information? %	How sufficient was the information? %
1 (very poor)	1.9	10.5
2 (poor)	11.3	18.5
3 (good)	45.3	37.6
4 (very good)	31.7	21.9
5 (excellent)	4.6	6.2
Missing	5.1	5.3
Total	100.0	100.0

The two independent physician reviewers selected the same underlying cause of death in 56.9% of the VAs, while consensus was reached after initial disagreement for 31.7%, while 10.0% required a panel decision to reach consensus, the remaining 1.3% were flagged for review by a maternal mortality specialist to exclude maternal deaths. In 68% of the deaths, the cause of death was coded to a usable code. Overall, 8.7% of the underlying COD were coded to ill-defined signs and symptoms, and 16.6% of the causes were considered to have insufficient specification within an ICD chapter.

**Figure 3.**
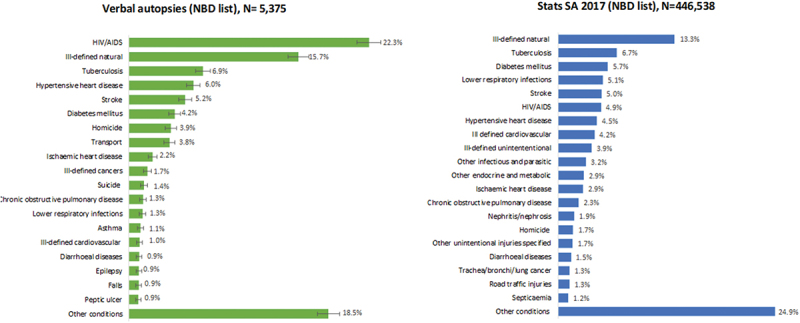
Leading causes of death based on 2017 stats SA data compared with physician reviewed verbal autopsy data aggregated to National Burden of disease list.

Figure 3 shows the leading causes of death from the physician reviews compared with vital statistics using the SA NBD list (Annexure 1, Table 1) [27]. A slightly higher proportion of the VAs (15.7%) were coded to ill-defined natural causes as compared to 13.3% of deaths in the Stats SA data. However, the ranking and proportions of the specified causes differed considerably. The VA identified 22.3% HIV/AIDS and 6.9% TB deaths, whereas in Stats SA data the HIV/AIDS and TB deaths accounted for 4.9% and 6.7%, respectively (Figure 3). According to WHO ICD-10 coding guidelines, TB deaths with co-morbid HIV are allocated to HIV/AIDS as the underlying cause, while TB deaths without co-morbid HIV are coded to TB as the underlying cause-of death (UCOD). Compared with the SA NBD study [29] albeit 5 years earlier, the VA profile appears more realistic than the Stats SA data, which are known to have extensive misattribution of HIV as a cause of death [30].

An additional feature of the VA data has been the use of 4-digit ICD-10 codes. This makes it possible to identify the deaths with underlying HIV disease that resulted in TB (B20.0)^1^^1^Stats SA coding of underlying cause does not code to 4-digits. which cannot be differentiated in the Stats SA data using the 3-digit ICD-10 codes. Almost half (49.3%) of the HIV/AIDS deaths (604/1 224) had associated tuberculosis, while 61.3% of the TB deaths had underlying HIV (604/984). These are important metrics for monitoring HIV and TB programmes.

From Figure 3, we also observe that the ranking of leading causes of non-communicable diseases differ

from Stats SA. The Stats SA data rank diabetes highest followed by stroke and hypertensive heart

disease, whereas it is the opposite for the VA data. The VA identified a slightly higher proportion of deaths from external causes than Stats SA (12.8% vs 11.2%).

**Figure 4.**
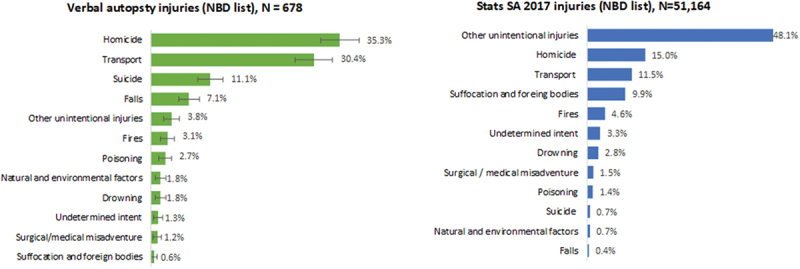
Leading injury-related causes of death stats SA, 2017 and physician reviewed verbal autopsy data aggregated to national burden of disease list.

Figure 4 shows different profiles of injury deaths. Homicide accounted for 35.3% of the VA deaths and only 15.0% of Stats SA causes. This appears to be related to the high proportion of ill-defined unintentional deaths (48.1%) in the Stats SA data shown in Figure 4. The ranking of the external causes of death based on the physician reviewed VAs aligns more closely with the SA NBD study [29] and the 2017 Injury Mortality Survey [31] which rank homicide highest, followed by transport and then suicide.

**Figure 5.**
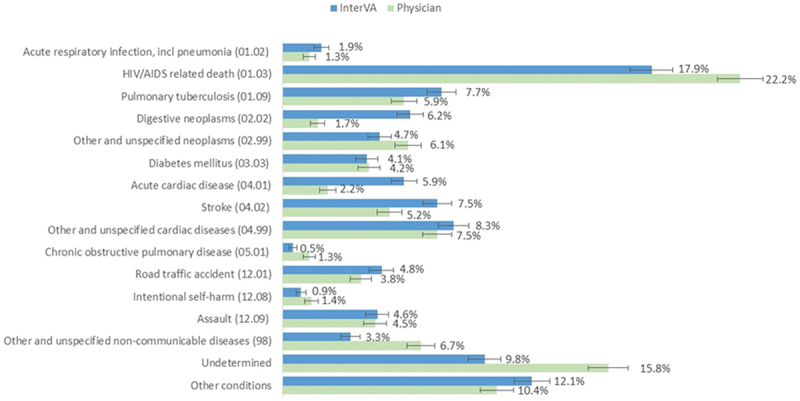
Comparison of verbal autopsy cause-specific mortality fractions based on physician reviews with InterVA-5 for the South African national cause of death validation project 2017/18.

CSMF derived by InterVA-5 and ranked to the top 15 causes from the VA list is compared with those from the physician coded VA in Figure 5. Although there is considerable similarity in the selection of the underlying COD, physicians assigned more cases to indeterminate underlying COD than the InterVA-5 tool, and there are some notable differences in the CSMF. Physicians identified lower proportions of acute cardiac disease and digestive neoplasms, and higher proportions of HIV/AIDS-related deaths, other unspecified non-communicable diseases, and other unspecified neoplasms compared with InterVA.

## Discussion

The study demonstrates that collecting COD data on a national scale using VA is achievable and provided good-quality COD information. The participation rate of next of kin was good once they had been identified and located, with 93.4% completed an interview. The physician reviewers found good-quality COD information was provided in 83% of the VAs. Of the identified COD, only 8.7% were coded to ill-defined natural causes and 68% were assigned a specific and valid underlying cause of death. The remaining 16.6% were assigned an underlying cause of death without sufficient specification. Although the sample cannot be considered nationally representative when compared with SA NBD estimates [29], the VAs provided a more realistic proportion of HIV/AIDS deaths and better information related to external causes of death than COD data from the CRVS system. Improved information related to injuries was obtained from the additional information provided by the narratives compared to Stats SA data.

The use of experienced interviewers was beneficial as it resulted in a very low refusal rate. Our experience of selecting fieldworkers was positive and is aligned with the criteria mentioned in the WHO manual for training of verbal autopsy interviewers. The educational level of VA interviewers varies across VA studies, however the impact of the educational background of VA interviewers on the quality of the VA information collected has not been formally investigated [32]. Some authors claim that accurate information can be collected by well-trained lay people from the community, whilst others advocate for medically trained interviewers [32,33]. Our choice of VA interviewers was influenced by the experience in HDSS in South Africa, where successful VA interviews have been conducted by lay people [17].

Recruiting the next of kin through funeral parlours and Home Affairs offices proved only partially effective due to the challenges posed by the Protection of Personal Information Act (POPIA) [20]. Under POPIA, the Departments of Home Affairs and Health cannot share next of kin information without consent. As a result, intermediaries, such as funeral practitioners and Home Affairs officials, were used to obtain consent. For national implementation of VA, a regulated process will be needed to identify deaths occurring outside of health facilities and to initiate contact with the next of kin.

The use of both physician review and an automated computerised model to ascertain probable COD provided similar results. However, physician review is time-consuming and costly. Variability in the identification of the underlying COD between physician reviewers further necessitates a review panel. Although automated computerised models such as InterVA are cheaper, faster, more consistent, and can be considered for routine coding of high volumes of VAs, quality assurance processes will still be important. Byass et al. [34] compared physician coded VA with InterVA-4 assigned COD from some African and Asian countries, and found strong concordance between physician coded VA and InterVA–4 assigned COD, however, they could not prove which approach provided the true cause of death [34].

Whilst the VA narrative is not used by the algorithms to assign a COD, clinicians find this information critical in determining the COD. In addition, the narrative provided an opportunity for the interviewer to establish rapport with the respondent, thus we feel strongly that the narrative be conducted at the beginning of the interview.

### Limitations

The COD patterns presented in this study cannot be assumed to be nationally representative because of the low sample realisation of (55%) VAs achieved. However, the sample does have national coverage, and the results are largely consistent with the national burden of disease profile. The potential bias due to poor sample realisation is not expected to have a major impact on the estimation of correction factors, which was the main objective of the project, however the extent and nature of potential biases remain unknown.

### Strengths

Quality of the information collected by the interviewers indicates the success of the training conducted by experienced researchers from Health and Demographic Surveillance Sites and a local research organisation. All the VA interviews were assessed by two independent physicians trained in medical certification of COD and how to interpret a VA. A systematic quality assurance process insured standardised interpretation of the VAs. The use of KoBoTool and other digital platforms enabled real-time monitoring of the fieldwork and the review of VAs.

## Conclusions

Despite challenges in recruiting the next of kin, our study has demonstrated the feasibility and community acceptability of conducting VAs to ascertain improved COD information. VA could be used at a national scale providing the recruitment of next of kin can be institutionalised into the routine processes for registration of death. It is expected that the use of VA will contribute information particularly for the deaths that occur outside health facilities. We recommend that specific questions in the 2016 WHO VA be clarified to make it easier for interviewers. Re-organising narratives to be conducted at the beginning of the interview worked well as a way of engaging the respondent and orientating the fieldworker.

## Supplementary Material

Annexure 1 .docx

## Data Availability

Not applicable in this section.

## Data deposition

Not applicable in this section.

## Appendix

### Annexure 1:

The basic NBD list (145 categories) is aligned with the SA NBD list but does not make any assumptions about misclassification of causes and has categories for ill-defined causes. Table A1 shows the ICD-10 codes for each category in the basic NBD list.

**Table A1.** ICD-10 codes for each category of the basic NBD list.

Basic NBD list		ICD-10 code
1	Tuberculosis	A15 - A19; U51 & U52; B90; J90
2	STD/excluding HIV	A50 - A64; N70 - N73
3	HIV/AIDS	B20 - B24; C46
4	Diarrhoeal diseases	A00 - A04; A06 - A09
5	Childhood (vaccine preventable) cluster	A33 - A37; A80; B03; B05; B06; B91
6	Bacterial meningitis	A39; G00; G03
7	Hepatitis	B15 - B19
8	Malaria	B50 - B54
9	Schistosomiasis and other tropical diseases	B55 - B56; B65; B74
10	Leprosy	A30; B92
11	Intestinal parasites	B76 - B81
12	Septicaemia	A40; A41
13	Other infectious and parasitic	A05; A20 – A28; A31; A32; A38; A42 – A49; A65 - A69; A70 - A74; A75 - A79; A81 - A89; A90 - A99; B00 - B02; B04; B07 - B09; B25 - B34; B35 - B49; B57 – B64; B66 – B73; B75; B82 – B89; B94 – B99
14	Lower respiratory infections	J09 - J18; J20 - J22
15	Upper respiratory infections	J00 - J06
16	Otitis media	H65; H66
17	Maternal haemorrhage	O20; O44 - O46; O67; O72
18	Maternal sepsis	O85
19	Hypertension in pregnancy	O10 - O16
20	Obstructed labour	O64 - O66
21	Abortion	O00 - O08
22	Other maternal	O21 - O29; O30 - O43; O47 - O48; O60 - O63; O68 - O71; O73 - O75; O80 - O84; O86 - O92; O95 - O99
23	Low birth weight	P05 - P07; P22
24	Birth asphyxia and trauma	P03; P10 - P15; P20 - P21
25	Other perinatal respiratory conditions	P23 - P29
26	Neonatal infections	P35 - P39
27	Other perinatal	P00 - P02; P04; P08; P29; P50 - P61; P70 - P94; P96
28	Ill-defined perinatal	P95
29	Protein-energy malnutrition	E40 - E46; D50 - D53; D64
31	Pellagra and other nutritional deficiencies	E00 - E02; E50 - E64
32	Mouth and oropharynx cancer	C00 - C14
33	Oesophagus cancer	C15
34	Stomach cancer	C16
35	Colo-rectal cancer	C18 - C21
36	Liver cancer	C22
37	Pancreas cancer	C25
38	Larynx cancer	C32
39	Trachea/bronchi/lung cancer	C33 - C34
40	Bone and connective tissue cancer	C40; C41; C47; C49
41	Melanoma of skin	C43
42	Other skin cancer	C44
43	Breast cancer	C50
44	Cervix cancer	C53
45	Corpus uteri cancer	C54; C55
46	Ovary cancer	C56
47	Prostate cancer	C61
48	Bladder cancer	C67
49	Kidney cancer	C64 - C66; C68
50	Brain cancer	C71
51	Lymphoma	C81 - C90; C96
52	Leukemia	C91 - C95
53	Other malignant neoplasms	C17; C23 - C24; C26; C30 - C31; C37 - C39; C45; C48; C51 - C52; C57 - C58; C60; C62 - C63; C69 - C70; C72 - C75
54	Ill-defined cancers	C76 - C80; C97
55	Benign neoplasms	D00 - D48
56	Diabetes mellitus	E10 - E14
57	Albinism	E70
58	Other endocrine and metabolic	D55 - D63; D65 - D89; E03 - E07; E15 - E16; E20 - E34; E65 - E68; E71 - E89
59	Alcohol dependence	F10
60	Drug use	F11 - F16; F18 - F19
61	Schizophrenia	F20 - F29
62	Unipolar	F32 - F33
63	Bipolar	F30 - F31
64	Anorexia Nervosa	F50
65	Obsessive compulsive/panic disorders	F40 - F42
66	Hyperkinetic disorders	F90
67	Adjustment reaction (PTSS)	F43
68	Mental disability	F70 - F79
69	Other mental disorders	F17; F34 - F39; F44 - F48; F51 - F59; F60 - F69; F80 - F89; F91 - F98; F99
70	Alzheimer and other dementias	G30 - G31; F01 - F09
71	Parkinson's disease	G20 - G21
72	Multiple sclerosis	G35
73	Epilepsy	G40 - G41
74	Encephalitis and brain abscess	G04; G06; G09
75	Other nervous system disorders	G08; G10 - G12; G23 - G25; G36 - G37; G36 - G37; G43 - G47; G50 - G58; G60 - G64; G70 - G72; G80 - G83; G90 - G98
76	Glaucoma	H40
77	Cataracts	H25 - H26
78	Other visual disorders	H00 - H21; H27 - H35; H42 - H59
79	Hearing loss and other ear disorders	H60 - H62; H68 - H95
80	Rheumatic heart disease	I01 - I09
81	Ischaemic heart disease	I20 - I25
82	Stroke	I60 - I69
83	Inflammatory heart disease	I30; I33; I38; I40; I42
84	Hypertensive heart disease	I10 - I13
85	Non-rheumatic valvular disease	I34 - I37
86	Pulmonary embolism	I26
87	Aortic aneurism	I71
88	Peripheral vascular disorders	I72 - I78; I80 - I84; I86 - I89;
89	Other cardiovascular	I00; I28; I31; I44 - I45; I95 - I99
90	Ill-defined cardio - heart failure etc	I46 - I49; I50 - I51; J81
91	Atherosclerosis	I70
92	Chronic obstructive pulmonary disease (COPD)	J40 - J44; I27
93	Asthma	J45 - J46
94	Aspiration pneumonia/lung abscess	J69; J85 - J86
95	Other respiratory	J30 - J39; J47; J60 - J68; J70; J80; J82 - J84; J92 - J98
96	Peptic ulcer	K25 - K28
97	Appendicitis	K35 - K37
98	Noninfective gastroenteritis and colitis	K50 - K52
99	Cirrhosis of liver	K70; K74; K76; I85
100	Hepatic failure	K72
101	Gall bladder disease	K80 - K83
102	Pancreatitis	K85; K86
103	Other digestive	K20 - K22; K29 - K31; K38; K40 - K46; K55; K66; K71; K73; K75; K90; K91
104	Ill-defined digestive	K92
105	Nephritis/nephrosis	N00 - N19
106	Benign prostatic hypertrophy	N40
107	Other genito-urinary	N20 - N23; N25 - N39; N41 - N50; N60 - N64; N75 - N98
108	Skin disease	L00 - L98
109	Rheumatoid arthritis	M05 - M06
110	Osteoarthritis	M15 - M19
111	Other musculo-skeletal	M00 - M02; M08; M10 - M13; M20 - M99
112	Neural tube defects	Q00 - Q07
113	Cleft lip/palate	Q35 - Q37
114	Congenital heart disease	Q20 - Q28
115	Congenital disorders of GIT	Q38 - Q45
116	Down syndrome and other chromosomal anomalies	Q90 - Q99
117	Foetal alcohol syndrome	Q86
118	Other congenital abnormalities	Q10 - Q18; Q30 - Q34; Q50 - Q56; Q60 - Q64; Q65 - Q79; Q80 - Q85; Q87
119	Ill-defined congenital	Q89
120	Dental caries	K02
121	Periodontal disease	K05
122	Other oral health	K00; K01; K03; K04; K06 - K14
123	Cot death	R95
124	Ill-defined natural	R00 - R09; R10 - R19; R20 - R23; R25 - R29; R30 - R39; R40 - R46; R47 - R49; R50 - R69; R70 - R79; R80 - R82; R83 - R94; R96 - R98; R99
125	Road traffic accidents	V01 - V04; V06; V09 - V80; V87; V89; V99
126	Non motor vehicle traffic accidents	V05; V81 - V86; V88; V90 - V94; V95 - V98
127	Mining accidents	Y37
128	Poisoning	X40 - X49
129	Surgical/medical misadventure	Y60 - Y69; Y70 - Y82; Y83 - Y84; Y88
130	Falls	W00 - W19
131	Fires	X00 - X09
132	Natural and environmental factors	W53 - W64; X20 - X29; X30 - X39; X50 - X57
133	Drowning	W65 - W74
134	Suffocation and foreign bodies	W75 - W84
135	Other unintentional injuries specified	W20 - W49; W50 - W52; W85 - W99; X10 - X19; X58; Y38; Y39; Y40 - Y59
136	Ill-defined transport	Y85
137	Ill-defined other unintentional	X59; Y86
138	Undetermined whether intentional or unintentional	Y10 - Y34; Y87; Y89
139	Suicide	X60 - X84
140	Homicide with firearm	X93 - X95
141	Homicide without firearm	X85 - X92; X96 - X99; Y00 -Y08
142	Ill-defined homicide	Y09
143	War	Y35; Y36

Table A2 provides the ICD-10 codes for the 64 categories in the VA list.

**Table A2.** ICD-10 codes for each category of the VA list.

VA List		ICD-10 code
101	Sepsis	A40 - A41
102	Acute respiratory infection, including pneumonia	J00 - J22
103	HIV/AIDS related death	B20 - B24
104	Diarrhoeal diseases	A00 - A09
105	Malaria	B50 - B54
106	Measles	B05
107	Meningitis and encephalitis	A39; G00 - G05
108	Tetanus	A33 - A35
109	Pulmonary tuberculosis	A15 - A16; U51 - U52
110	Pertussis	A37
111	Haemorrhagic fever	A92 - A99
112	Dengue fever	A91
199	Other and unspecified infectious disease	A17 - A19; A20 - A38; A42 - A44; A46; A48 - A89; B00 - B19; B25- B49; B55 - B99
201	Oral neoplasms	C00 - C06
202	Digestive neoplasms	C15 - C26
203	Respiratory neoplasms	C30 - C39
204	Breast neoplasms	C50
205	Female reproductive neoplasms	C51 - C58
206	Male reproductive neoplasms	C60 - C63
299	Other and unspecified neoplasms	C07 - C14; C40 - C49; C60 - D48
301	Severe anaemia	D50 - D64
302	Severe malnutrition	E40 - E46
303	Diabetes mellitus	E10 - E14
401	Acute cardiac disease	I20 - I25
402	Stroke	I60 - I69
403	Sickle cell with crisis	D57
499	Other and unspecified cardiac disease	I00 - I09; I10 - I15; I26 - I52; I70 - I99
501	Chronic obstructive pulmonary disease (COPD)	J40 - J44
502	Asthma	J45 - J46
601	Acute abdomen	K35 - K37; K40 - K46; K56; R10
602	Liver cirrhosis	K70 - K76
701	Renal failure	N17 - N19
801	Epilepsy	G40 - G41
9800	Other and unspecified non-communicable disease	D55 - D89; E00 - E07; E15 - E35; E50 - E90; F00 - F99; G06 - G09; G10 - G37; G43 - G47; G50 - G99; H00- H95; J30 - J39; J47 - J99; K00 - K31; K35- K38; K40 - K93; L00 - L99; M00 - M99; N00- N16; N20 - N99; R00 - R09; R11 - R94
901	Ectopic pregnancy	O00
902	Abortion-related death	O03 - O08
903	Pregnancy-induced hypertension	O10 - O16
904	Obstetric haemorrhage	O46; O67; O72
905	Obstructed labour	O63; O66
906	Pregnancy-related sepsis	O85
907	Anaemia of pregnancy	O99
908	Ruptured uterus	O71
999	Other and unspecified maternal cause	O01 - O02; O20 - O45; O47 - O62; O68 - O70; O73 - O75; O76 - O84; O86 - O98
1001	Prematurity	P05 - P07
1002	Birth asphyxia	P20 - P22
1003	Neonatal pneumonia	P23 - P25
1004	Neonatal sepsis	P36
1005	Neonatal tetanus	A33
1006	Congenital malformation	Q00 - Q99
1099	Other and unspecified perinatal cause of death	P00 - P04; P08 - P15; P26 - P35; P37 - P94; P96
1100	Stillbirths	P95
1201	Road traffic accident	V01 - V89
1202	Other transport accident	V90 - V99
1203	Accidental fall	W00 - W19
1204	Accidental drowning and submersion	W65 - W74
1205	Accidental exposure to smoke, fire and flames	X00 - X19
1206	Contact with venomous animals and plants	X20 - X29
1207	Accidental poisoning and exposure to noxious substance	X40 - X49
1208	Intentional self-harm	X60 - X84
1209	Assault	X85 - Y09
1210	Exposure to force of nature	X30 - X39
1299	Other and unspecified external cause of death	S00 - T99; W20 - W64; W75 - W99; X50 - X59; Y10 - Y98
9900	Cause of death unknown	R95 - R99
