# Supplementary material for: Can verbal autopsies be used on a national scale? Key findings and lessons from South Africa's national cause-of-death validation study
Source: Glob Health Action. 2024 Sep 13;17(1):2399413. doi: 10.1080/16549716.2024.2399413 (PMC11404373; doi:10.1080/16549716.2024.2399413)
Supplement: Annexure 1 .docx [file ZGHA_A_2399413_SM5980.docx]

**Annexure1: Basic NBD list and VA list**

The basic NBD list (145 categories) is aligned with the SA NBD list but does not make any assumptions about misclassification of causes and has categories for ill-defined causes. Table 1 shows the ICD-10 codes for each category in the basic NBD list.

Table 1: ICD-10 codes for each category of the basic NBD list.

| **Basic NBD list** | | **ICD-10 code** |
| --- | --- | --- |
| 1 | Tuberculosis | A15 - A19; U51 & U52; B90; J90 |
| 2 | STD/excluding HIV | A50 - A64; N70 - N73 |
| 3 | HIV/AIDS | B20 - B24; C46 |
| 4 | Diarrhoeal diseases | A00 - A04; A06 - A09 |
| 5 | Childhood (vaccine preventable) cluster | A33 - A37; A80; B03; B05; B06; B91 |
| 6 | Bacterial meningitis | A39; G00; G03 |
| 7 | Hepatitis | B15 - B19 |
| 8 | Malaria | B50 - B54 |
| 9 | Schistosomiasis and other tropical diseases | B55 - B56; B65; B74 |
| 10 | Leprosy | A30; B92 |
| 11 | Intestinal parasites | B76 - B81 |
| 12 | Septicaemia | A40; A41 |
| 13 | Other infectious and parasitic | A05; A20 – A28; A31; A32; A38; A42 – A49; A65 - A69; A70 - A74; A75 - A79; A81 - A89; A90 - A99; B00 - B02; B04; B07 - B09; B25 - B34; B35 - B49; B57 – B64; B66 – B73; B75; B82 – B89; B94 – B99 |
| 14 | Lower respiratory infections | J09 - J18; J20 - J22 |
| 15 | Upper respiratory infections | J00 - J06 |
| 16 | Otitis media | H65; H66 |
| 17 | Maternal haemorrhage | O20; O44 - O46; O67; O72 |
| 18 | Maternal sepsis | O85 |
| 19 | Hypertension in pregnancy | O10 - O16 |
| 20 | Obstructed labour | O64 - O66 |
| 21 | Abortion | O00 - O08 |
| 22 | Other maternal | O21 - O29; O30 - O43; O47 - O48; O60 - O63; O68 - O71; O73 - O75; O80 - O84; O86 - O92; O95 - O99 |
| 23 | Low birth weight | P05 - P07; P22 |
| 24 | Birth asphyxia and trauma | P03; P10 - P15; P20 - P21 |
| 25 | Other perinatal respiratory conditions | P23 - P29 |
| 26 | Neonatal infections | P35 - P39 |
| 27 | Other perinatal | P00 - P02; P04; P08; P29; P50 - P61; P70 - P94; P96 |
| 28 | Ill-defined perinatal | P95 |
| 29 | Protein-energy malnutrition | E40 - E46; D50 - D53; D64 |
| 31 | Pellagra and other nutritional deficiencies | E00 - E02; E50 - E64 |
| 32 | Mouth and oropharynx cancer | C00 - C14 |
| 33 | Oesophagus cancer | C15 |
| 34 | Stomach cancer | C16 |
| 35 | Colo-rectal cancer | C18 - C21 |
| 36 | Liver cancer | C22 |
| 37 | Pancreas cancer | C25 |
| 38 | Larynx cancer | C32 |
| 39 | Trachea/bronchi/lung cancer | C33 - C34 |
| 40 | Bone and connective tissue cancer | C40; C41; C47; C49 |
| 41 | Melanoma of skin | C43 |
| 42 | Other skin cancer | C44 |
| 43 | Breast cancer | C50 |
| 44 | Cervix cancer | C53 |
| 45 | Corpus uteri cancer | C54; C55 |
| 46 | Ovary cancer | C56 |
| 47 | Prostrate cancer | C61 |
| 48 | Bladder cancer | C67 |
| 49 | Kidney cancer | C64 - C66; C68 |
| 50 | Brain cancer | C71 |
| 51 | Lymphoma | C81 - C90; C96 |
| 52 | Leukemia | C91 - C95 |
| 53 | Other malignant neoplasms | C17; C23 - C24; C26; C30 - C31; C37 - C39; C45; C48; C51 - C52; C57 - C58; C60; C62 - C63; C69 - C70; C72 - C75 |
| 54 | Ill-defined cancers | C76 - C80; C97 |
| 55 | Benign neoplasms | D00 - D48 |
| 56 | Diabetes mellitus | E10 - E14 |
| 57 | Albinism | E70 |
| 58 | Other endocrine and metabolic | D55 - D63; D65 - D89; E03 - E07; E15 - E16; E20 - E34; E65 - E68; E71 - E89 |
| 59 | Alcohol dependence | F10 |
| 60 | Drug use | F11 - F16; F18 - F19 |
| 61 | Schizophrenia | F20 - F29 |
| 62 | Unipolar | F32 - F33 |
| 63 | Bipolar | F30 - F31 |
| 64 | Anorexia Nervosa | F50 |
| 65 | Obsessive compulsive/ panic disorders | F40 - F42 |
| 66 | Hyperkinetic disorders | F90 |
| 67 | Adjustment reaction (PTSS) | F43 |
| 68 | Mental disability | F70 - F79 |
| 69 | Other mental disorders | F17; F34 - F39; F44 - F48; F51 - F59; F60 - F69; F80 - F89; F91 - F98; F99 |
| 70 | Alzheimer and other dementias | G30 - G31; F01 - F09 |
| 71 | Parkisons disease | G20 - G21 |
| 72 | Multiple sclerosis | G35 |
| 73 | Epilepsy | G40 - G41 |
| 74 | Encephalitis and brain abscess | G04; G06; G09 |
| 75 | Other nervous system disorders | G08; G10 - G12; G23 - G25; G36 - G37; G36 - G37; G43 - G47; G50 - G58; G60 - G64; G70 - G72; G80 - G83; G90 - G98 |
| 76 | Glaucoma | H40 |
| 77 | Cataracts | H25 - H26 |
| 78 | Other visual disorders | H00 - H21; H27 - H35; H42 - H59 |
| 79 | Hearing loss and other ear disorders | H60 - H62; H68 - H95 |
| 80 | Rheumatic heart disease | I01 - I09 |
| 81 | Ischaemic heart disease | I20 - I25 |
| 82 | Stroke | I60 - I69 |
| 83 | Inflammatory heart disease | I30; I33; I38; I40; I42 |
| 84 | Hypertensive heart disease | I10 - I13 |
| 85 | Non-rheumatic valvular disease | I34 - I37 |
| 86 | Pulmonary embolism | I26 |
| 87 | Aortic aneurism | I71 |
| 88 | Peripheral vascular disorders | I72 - I78; I80 - I84; I86 - I89; |
| 89 | Other cardiovascular | I00; I28; I31; I44 - I45; I95 - I99 |
| 90 | Ill-defined cardio - heart failure etc | I46 - I49; I50 - I51; J81 |
| 91 | Atherosclerosis | I70 |
| 92 | Chronic obstructive pulmonary disease (COPD) | J40 - J44; I27 |
| 93 | Asthma | J45 - J46 |
| 94 | Aspiration pneumonia/ lung abscess | J69; J85 - J86 |
| 95 | Other respiratory | J30 - J39; J47; J60 - J68; J70; J80; J82 - J84; J92 - J98 |
| 96 | Peptic ulcer | K25 - K28 |
| 97 | Appendicitis | K35 - K37 |
| 98 | Noninfective gastroenteritis and colitis | K50 - K52 |
| 99 | Cirrhosis of liver | K70; K74; K76; I85 |
| 100 | Hepatic failure | K72 |
| 101 | Gall bladder disease | K80 - K83 |
| 102 | Pancreatitis | K85; K86 |
| 103 | Other digestive | K20 - K22; K29 - K31; K38; K40 - K46; K55; K66; K71; K73; K75; K90; K91 |
| 104 | Ill-defined digestive | K92 |
| 105 | Nephritis/nephrosis | N00 - N19 |
| 106 | Benign prostatic hypertrophy | N40 |
| 107 | Other genito-urinary | N20 - N23; N25 - N39; N41 - N50; N60 - N64; N75 - N98 |
| 108 | Skin disease | L00 - L98 |
| 109 | Rheumatoid arthritis | M05 - M06 |
| 110 | Osteoarthritis | M15 - M19 |
| 111 | Other musculo-skeletal | M00 - M02; M08; M10 - M13; M20 - M99 |
| 112 | Neural tube defects | Q00 - Q07 |
| 113 | Cleft lip/palate | Q35 - Q37 |
| 114 | Congenital heart disease | Q20 - Q28 |
| 115 | Congenital disorders of GIT | Q38 - Q45 |
| 116 | Down syndrome and other chromosomal anomalies | Q90 - Q99 |
| 117 | Fetal alcohol syndrome | Q86 |
| 118 | Other congenital abnormalities | Q10 - Q18; Q30 - Q34; Q50 - Q56; Q60 - Q64; Q65 - Q79; Q80 - Q85; Q87 |
| 119 | Ill-defined congenital | Q89 |
| 120 | Dental caries | K02 |
| 121 | Periodontal disease | K05 |
| 122 | Other oral health | K00; K01; K03; K04; K06 - K14 |
| 123 | Cot death | R95 |
| 124 | Ill-defined natural | R00 - R09; R10 - R19; R20 - R23; R25 - R29; R30 - R39; R40 - R46; R47 - R49; R50 - R69; R70 - R79; R80 - R82; R83 - R94; R96 - R98; R99 |
| 125 | Road traffic accidents | V01 - V04; V06; V09 - V80; V87; V89; V99 |
| 126 | Non motor vehicle traffic accidents | V05; V81 - V86; V88; V90 - V94; V95 - V98 |
| 127 | Mining accidents | Y37 |
| 128 | Poisoning | X40 - X49 |
| 129 | Surgical / medical misadventure | Y60 - Y69; Y70 - Y82; Y83 - Y84; Y88 |
| 130 | Falls | W00 - W19 |
| 131 | Fires | X00 - X09 |
| 132 | Natural and environmental factors | W53 - W64; X20 - X29; X30 - X39; X50 - X57 |
| 133 | Drowning | W65 - W74 |
| 134 | Suffocation and foreign bodies | W75 - W84 |
| 135 | Other unintentional injuries specified | W20 - W49; W50 - W52; W85 - W99; X10 - X19; X58; Y38; Y39; Y40 - Y59 |
| 136 | Ill-defined transport | Y85 |
| 137 | Ill-defined other unintentional | X59; Y86 |
| 138 | Undetermined whether intentional or unintentional | Y10 - Y34; Y87; Y89 |
| 139 | Suicide | X60 - X84 |
| 140 | Homicide with firearm | X93 - X95 |
| 141 | Homicide without firearm | X85 - X92; X96 - X99; Y00 -Y08 |
| 142 | Ill-defined homicide | Y09 |
| 143 | War | Y35; Y36 |

Table 2 provides the ICD-10 codes for the 64 categories in the VA list.

Table 2: ICD-10 codes for each category of the VA list.

| **VA List** | | **ICD-10 code** |
| --- | --- | --- |
| 101 | Sepsis | A40 - A41 |
| 102 | Acute respiratory infection, including pneumonia | J00 - J22 |
| 103 | HIV/AIDS related death | B20 - B24 |
| 104 | Diarrhoeal diseases | A00 - A09 |
| 105 | Malaria | B50 - B54 |
| 106 | Measles | B05 |
| 107 | Meningitis and encephalitis | A39; G00 - G05 |
| 108 | Tetanus | A33 - A35 |
| 109 | Pulmonary tuberculosis | A15 - A16; U51 - U52 |
| 110 | Pertussis | A37 |
| 111 | Haemorrhagic fever | A92 - A99 |
| 112 | Dengue fever | A91 |
| 199 | Other and unspecified infectious disease | A17 - A19; A20 - A38; A42 - A44; A46; A48 - A89; B00 - B19; B25- B49; B55 - B99 |
| 201 | Oral neoplasms | C00 - C06 |
| 202 | Digestive neoplasms | C15 - C26 |
| 203 | Respiratory neoplasms | C30 - C39 |
| 204 | Breast neoplasms | C50 |
| 205 | Female reproductive neoplasms | C51 - C58 |
| 206 | Male reproductive neoplasms | C60 - C63 |
| 299 | Other and unspecified neoplasms | C07 - C14; C40 - C49; C60 - D48 |
| 301 | Severe anaemia | D50 - D64 |
| 302 | Severe malnutrition | E40 - E46 |
| 303 | Diabetes mellitus | E10 - E14 |
| 401 | Acute cardiac disease | I20 - I25 |
| 402 | Stroke | I60 - I69 |
| 403 | Sickle cell with crisis | D57 |
| 499 | Other and unspecified cardiac disease | I00 - I09; I10 - I15; I26 - I52; I70 - I99 |
| 501 | Chronic obstructive pulmonary disease (COPD) | J40 - J44 |
| 502 | Asthma | J45 - J46 |
| 601 | Acute abdomen | K35 - K37; K40 - K46; K56; R10 |
| 602 | Liver cirrhosis | K70 - K76 |
| 701 | Renal failure | N17 - N19 |
| 801 | Epilepsy | G40 - G41 |
| 9800 | Other and unspecified non-communicable disease | D55 - D89; E00 - E07; E15 - E35; E50 - E90; F00 - F99; G06 - G09; G10 - G37; G43 - G47; G50 - G99; H00- H95; J30 - J39; J47 - J99; K00 - K31; K35- K38; K40 - K93; L00 - L99; M00 - M99; N00- N16; N20 - N99; R00 - R09; R11 - R94 |
| 901 | Ectopic pregnancy | O00 |
| 902 | Abortion-related death | O03 - O08 |
| 903 | Pregnancy-induced hypertension | O10 - O16 |
| 904 | Obstetric haemorrhage | O46; O67; O72 |
| 905 | Obstructed labour | O63; O66 |
| 906 | Pregnancy-related sepsis | O85 |
| 907 | Anaemia of pregnancy | O99 |
| 908 | Ruptured uterus | O71 |
| 999 | Other and unspecified maternal cause | O01 - O02; O20 - O45; O47 - O62; O68 - O70; O73 - O75; O76 - O84; O86 - O98 |
| 1001 | Prematurity | P05 - P07 |
| 1002 | Birth asphyxia | P20 - P22 |
| 1003 | Neonatal pneumonia | P23 - P25 |
| 1004 | Neonatal sepsis | P36 |
| 1005 | Neonatal tetanus | A33 |
| 1006 | Congenital malformation | Q00 - Q99 |
| 1099 | Other and unspecified perinatal cause of death | P00 - P04; P08 - P15; P26 - P35; P37 - P94; P96 |
| 1100 | Stillbirths | P95 |
| 1201 | Road traffic accident | V01 - V89 |
| 1202 | Other transport accident | V90 - V99 |
| 1203 | Accidental fall | W00 - W19 |
| 1204 | Accidental drowning and submersion | W65 - W74 |
| 1205 | Accidental exposure to smoke, fire and flames | X00 - X19 |
| 1206 | Contact with venomous animals and plants | X20 - X29 |
| 1207 | Accidental poisoning and exposure to noxious substance | X40 - X49 |
| 1208 | Intentional self-harm | X60 - X84 |
| 1209 | Assault | X85 - Y09 |
| 1210 | Exposure to force of nature | X30 - X39 |
| 1299 | Other and unspecified external cause of death | S00 - T99; W20 - W64; W75 - W99; X50 - X59; Y10 - Y98 |
| 9900 | Cause of death unknown | R95 - R99 |
